# The Place of Gender Stereotypes in the Network of Cognitive Abilities, Self-Perceived Ability and Intrinsic Value of School in School Children Depending on Sex and Preferences in STEM

**DOI:** 10.3390/bs12030075

**Published:** 2022-03-10

**Authors:** Victoria Ismatullina, Timofey Adamovich, Ilya Zakharov, Georgy Vasin, Ivan Voronin

**Affiliations:** 1 Psychological Institute of Russian Academy of Education, 125009 Moscow, Russia; tadamovich11@gmail.com (T.A.); iliazaharov@gmail.com (I.Z.); georgy.vasin@gmail.com (G.V.); 2 Faculté des Sciences Sociales, École de Psychologie, Université Laval, Quebec City, QC G1V 0A6, Canada; ivan.a.voronin@gmail.com

**Keywords:** STEM, gender stereotypes, non-verbal intelligence, spatial abilities, self-perceived ability, academic motivation, math, science, school children, network structure

## Abstract

Adolescents face many barriers on the path towards a STEM profession, especially girls. We examine the gender stereotypes, cognitive abilities, self-perceived ability and intrinsic values of 546 Russian school children from 12 to 17 years old by sex and STEM preferences. In our sample, STEM students compared to no-STEM have higher cognitive abilities, intrinsic motivation towards math and science, are more confident in their math abilities and perceive math as being easier. Boys scored higher in science, math and overall academic self-efficacy, intrinsic learning motivation and math’s importance for future careers. Meanwhile, girls displayed higher levels of gender stereotypes related to STEM and lower self-efficacy in math. A network analysis was conducted to identify the structure of psychological traits and the position of the stem-related stereotypes among them. The analysis arrived at substantially different results when adolescents were grouped by sex or preference towards STEM. It also demonstrated that gender stereotypes are connected with cognitive abilities, with a stronger link in the no-STEM group. Such stereotypes play a more important role for girls than boys and, jointly with the general self-efficacy of cognitive and academic abilities, are associated with the factors that distinguish groups of adolescents in their future careers.

## 1. Introduction

Maintaining the high level of human capital is one of the main challenges faced by any society that strives for success in the 21st century. The availability of a sufficient number of highly skilled specialists in the areas of science, technology, engineering and mathematics (STEM) is particularly important for rapid technological and economic development. Therefore, enrolling adolescents in STEM disciplines and providing STEM tracks at all levels of education are top priorities for the educational system in any modern technological society.

Many educators put forth an idea that choosing a career is a continuous process that can span a lifetime; however, the educational and psychosocial experiences in middle and high school are considered to be decisive for one’s career choice [1]. It is during adolescence that the career aspirations emerge from the individual’s abilities, interests and values to later determine the academic and career choice in STEM [2,3].

The process of choosing a career in Russia is rarely accompanied by any professional guidance. Academic counselors/tutors are rare in academic institutions in Russia, and the decision to choose a career mainly takes place within families. At the same time, from the point of transferring a set of knowledge about a future career in Russia, schools currently cannot cope with transferring an up-to-date picture to schoolchildren: according to a study by Savostina, Smirnova and Khasbulatova [4], of those who would like to work in the STEM field in the future (47%), 92% noted a general interest in science and technology-related issues and 74% an interest in related professions, but only 47% said they received information about such professions in school. In general, school lessons remain an underutilized resource for shaping the choice of a professional future. According to surveys on choosing a career in STEM areas [4], up to half of the surveyed schoolchildren note a lack of objective information about the prospects for the profession market in Russia, and in the world, in terms of schools giving help in choosing a profession, high school students of both sexes ranked teachers 10th out of 16. The process of choosing a career, including STEM careers, is often described in the framework of the social cognitive career theory (SCCT) [5,6]. In its application to STEM careers, SCCT distinguishes the following key factors that determine career choice:

- mathematical achievement in high school;
- engagement with STEM disciplines at school;
- mathematical self-efficacy (one’s beliefs about their ability to solve mathematical problems), supported by previous achievement and attitude towards mathematics;
- intention to specialize in STEM, including extracurricular activities and outcome expectations.

Previous studies have shown that students who choose STEM as their future career demonstrate higher performance in cognitive tests compared to their non-STEM peers [7,8]. However, although SCCT identifies the level of cognitive ability as a crucial prerequisite for the skills and knowledge in math and science required for STEM professions, the authors emphasize that it does not necessarily mean that the person with high cognitive ability or advanced skills in math and science will engage in STEM-related activities or pursue a STEM career. Some authors point out that the ability to maintain motivation is another important component of a successful career path since it allows continuous use and development of abilities and skills necessary for STEM careers (e.g., [9,10]). Therefore, SCCT concentrates on the core beliefs, interests and values that an individual attaches to their area of academic and professional specialization that can be as important for the career choice as cognitive ability or academic skills.

Psychometric intelligence, or general cognitive ability, is one of the most important sources of the individual differences in learning performance [11,12]. It is a powerful predictor of the achievement across different academic domains [13,14] and individual behavior; in particular, the strategic behavior required for day-to-day decision-making processes [15]. Cognitive abilities are also associated with wages, education and employment [16]. The general cognitive ability level may also be related not only with day-to-day decisions but to more general questions such as career choice. This notion is indirectly supported by the reported positive correlation between IQ and one’s average income level [17,18]. Spatial ability is another important cognitive predictor of academic success in STEM. Although it partly overlaps with intelligence, it maintains its contribution to academic achievement even after verbal and mathematical reasoning are controlled for [7]. Spatial ability is involved in many everyday activities, such as navigation on the streets or parking the car [19], but it is also a strong predictor of school achievement in STEM subjects, specifically, math [20]. Well-developed spatial reasoning is important for professions such as architecture, engineering and computer science [19,21]. Beliefs about one’s own abilities, or self-efficacy, can affect the interest and perception of one’s aptitude towards STEM. Self-efficacy is grounded in the student’s actual academic achievement since the process of learning is guided by the feedback about the student’s performance. At the same time, a big part of the differences in self-efficacy cannot be explained by actual academic achievement. Specifically, when gender groups are compared at the same level of cognitive abilities, 44.2% of girls doubt their attitude towards STEM disciplines and 58.1% assume that they have no abilities for these subjects, while only 23–25% of the boy’s express similar concerns. When asked about the motivation to choose a particular academic specialization, 59.7% of girls respond that they are guided by the level of their abilities, rather than by the prestige and profitability of the profession [4]. The studies show that the relatively low mathematical self-efficacy in girls predicts lower aspirations for pursuing mathematically-loaded careers [22]. The expectation of success, self-confidence and self-efficacy are important predictors of the future career choice [9,23,24]. The expectation of success varies across the subject domains, and individuals usually choose the occupation that they expect to succeed at [25]. The students with a higher appraisal of their mathematical competence more often enroll in advanced mathematical courses, choose quantitative specialization in college and start a career in STEM [26]. At the same time, male high school students tend to give higher appraisal of their mathematical competence, compared to their female counterparts with similar math grades [27]. It is important to admit that although self-efficacy plays an important role in career choice, it does not provide the full account of the academic or career choice [28,29,30]. Students’ research experience and proficiency in mathematics and science also predict success in STEM [31,32]. Some studies that addressed the effect of extracurricular activities and person–environment fit in secondary school on the choice of main academic specialization [33,34,35] found that engaging in math and science courses in high school encourages students to apply for and pursue STEM degrees. In other studies, GPA (Grade Point Average), academic self-efficacy and the availability of training courses were identified as the factors of choice of academic specialization [3,34]. Despite the fact that family and school contexts, particularly peers, affect academic achievement and motivation, the research into the role of peers in the choice of academic specialization is lacking. Recent studies show that girls whose friends value achievement in math and science, underestimate or devalue the importance of English and vice versa [36]. Generally, the understanding of STEM as the key academic disciplines for economic development is universally accepted in economically successful countries, for example, in the Asia Pacific countries. At the same time, many of these countries maintain the local stereotypes about races and socio-economic groups [33,34,37,38,39]. Despite the noticeable cross-cultural differences between the countries, it is especially concerning that the most common obstacle in a career choice in nearly all these countries is gender stereotypes. The STEM-related gender stereotypes manifest in various ways. For example, some people believe that women’s gender roles do not include a STEM career because it contradicts their duty to take care of the house. Another powerful stereotype is that boys outperform girls in mathematical tasks, and, accordingly, should be more successful in STEM. Such under-estimation of girls’ potential can often be expressed by parents or school teachers who may persuade girls not to enter STEM education. At the same time, girls who have high achievements in STEM disciplines may fear that they may feel lonely in STEM without same-gender peers and that they will face discrimination by employers in the future.

The data show that fewer women than men work in STEM professions, and their salary is lower. For example, women only make up 21% of IT specialists with a university degree, earning 82.6% of the average man’s salary in the same position. Among technicians, only 24.2% are women, earning 68.3% of the average man’s salary [4]. According to a statistical report from 2015, in Russia, women make up 29% of the high-level specialists in the field of natural and exact sciences and 25% of the mid-level specialists in physics and engineering. As a reference, in health care, biology and agriculture, 69% of the high-level specialists are women. Gender imbalance in STEM professions and strict gender roles in Russian society reinforce the gender stereotypes, resulting in a gender gap. The gender gap in STEM is a condition when girls have less interest in STEM professions, choose them less often and have less success, compared to their male peers [40,41].

At the end of 2018, the Ministry of Education of Russia enacted a new conception of technological education that established a network of technological workshops across Russia aiming to provide Russian school students with an enriched educational environment. Since 2019, the project “Women in digital economy. A STEM project” has been communicating the knowledge about the role of women in socio-economic development to the government, business and professional associations in Russia. Despite the fact that, according to the Global Gender Gap report (Educational Attainment score), Russia has a high level of women’s involvement in education (24th place across the globe), women choose the STEM professions less often than men, and those who do choose these professions earn less or are hindered in their career progress (“glass ceiling”). In other words, women are poorly represented in STEM and practically absent in the sphere of strategic decisions [42,43]. The surveys of university applicants show that despite high academic achievement in STEM subjects, girls prefer not to choose STEM specializations for higher education [44]. The effect of gender stereotypes on academic motivation arise from social roles and rules regarding gender that dictate behavior in social contexts, including career choice. Girls’ lack of interest and low self-appraisal in math and science is a reason why girls less frequently engage in activities related to math and science, and rarely choose these or other STEM subjects for advanced study [45,46,47]. Gender disparity in STEM may be related to gender stereotypes and to the difference in perceived value of math for girls and boys [48,49]. The low involvement in STEM among girls can also be associated with the stereotypes about traditionally defined male and female roles. Compared to boys, girls tend to underestimate their abilities in math even when they have a similar level of academic achievement [50,51,52,53]. This situation is typical for students in both primary school [54] and secondary or high school [55]. Girls are also less interested in math and consider it as less important for the future [56,57]. Boys are more focused on achievement in mathematics and are more likely to choose it for advanced study [45,46,58]. Parent and teacher support is especially important for girls because their self-efficacy in STEM disciplines is guided more by others’ opinions, rather than their positive experiences [59,60,61].

In addition to the family environment and peer perceptions, one of the most important sources of career attitudes is also the attitudes transmitted to children by their teachers. For example, studies on the attitudes of primary and secondary school teachers have shown their negative attitudes towards science and technology [62]. Teachers’ self-perception, confidence and competency in teaching science can determine the format of learning. In primary education, the fact that many teachers (primarily female) feel insecure and a lack of proficiency in STEM-related activities can lead to more strict pedagogical practices that make it difficult for students to learn, enjoy and develop an interest in exploring these areas more deeply [63,64]. Moreover, negative experiences of the subject matter can lead to the formation of specific types of anxiety (e.g., mathematical anxiety), which in turn can affect both learning success and subsequent choices [65].

The appraisal of one’s own mathematical achievement differs between boys and girls as early as in primary school: girls believe that they perform worse in math in comparison with boys [66]. The effect of gender stereotypes on professional activities and career is well-researched [67]. Interestingly, girls’ achievement is affected by beliefs of their parents, specifically, their mothers, who believe that boys do not have an innate advantage over girls in mathematical abilities, to protect their daughters from a harmful effect of the stereotype threat [68].

Abilities, academic achievement, motivation and stereotypic beliefs make a complex system of intertwined individual traits that drive the career choice. One of the promising ways to investigate the structure of connections between the psychological characteristics is network analysis. The network analysis approaches the system as a whole and analyzes the components of the system as well as relations between them. The common examples of networks are transport networks [69] and social networks [70]. The common belief behind the network analysis states that the structure of psychological constructs is better described with the emergent properties of complex systems rather than latent psychological factors [71]. The topology of the network is believed to be a better description of complex psychological phenomena than a number of pairwise interactions between the patterns [72].

In networks of psychological traits, nodes represent observed variables, and edges represent the strength of associations between two variables [73]. According to the network science perspective, the psychological constructs are not the single entities but rather a set of interconnected components, where one part of the system can trigger another part of the system, which, in turn, triggers the third part and so on. The network analysis has been recently used in the studies of psychopathology [73], psychological traits [72] and relations between psychological and environmental factors [74]. The network analysis allows researchers to investigate the place of the specific traits in the topology of the network of the psychological traits and connect correlations to causations [73]. Thus, network analysis has become a valuable tool in psychological research.

Overall, adolescents face many barriers on the path towards a STEM profession, especially girls. Our study aims to examine gender stereotypes, cognitive abilities, self-perceived ability and the intrinsic values of Russian adolescents who chose STEM for specialization, regardless of gender, to determine the differences in the trait levels between the two groups. Based on previous research, we compare whether the adolescents who chose a STEM specialization differ from their peers in terms of their cognitive abilities and perceptions about STEM. Additionally, we compare boys and girls in terms of these characteristics. We hypothesize that boys will score higher in the traits that are relevant for STEM, and girls will show more gender stereotypes about STEM. The main goal of our study is to describe the structure of relationships between the traits in the STEM/non-STEM and boys/girls groups and to determine whether STEM-related gender stereotypes have a special place among other factors affecting future career choices.

## 2. Materials and Methods

To test the study hypothesis, we first divided our sample into two large groups, according to the preferences of adolescents in choosing the direction of their future career: STEM and non-STEM. This allowed us to test the hypothesis that adolescents from the STEM group differ in the declared characteristics from adolescents who chose a different direction. After that, we compared the groups by sex of participants according to the same indicators in order to assess the presence of differences in these groups. In order to answer the main question of our study regarding the place of the gender gap in the structure of factors affecting STEM-related career choices, we conducted a network analysis.

### 2.1. Participants

Participants of the study were contacted through the schools that they attended. The adolescents’ parents or guardians as well as the school administrations granted us their informed consent for administering our battery of tests to the participants on school grounds. Due to the restrictions associated with COVID-19, all the assessment took place online: part of the assessment was administered during an online school lesson, while some students participated in the assessment online on their own. All tests were administered on personal computers using an online platform (https://digitalpsytools.ru/ (accessed on 12 August 2021)). This platform can be used to create a test battery and generate a link for participants, as well as a list of unique IDs and passwords for each participant. Importantly, this platform enabled participants to pause the test at any moment and resume it later. The whole assessment took roughly one school lesson, 45 min. At any moment, participants could contact an assessment advisor to ask any questions or resolve difficulties. Students were not rewarded in a tangible way for participating in the sudy. The study was conducted according to the guidelines of the Declaration of Helsinki. The methodology was approved by the Ethics Committee of the Psychological Institute of the Russian Academy of Education.

Our sample consisted of 546 Russian pupils of secondary schools from 12 to 17 years old (mean 14.6, SD = 1.34.; 273 boys and 273 girls), these remained from the 615 initial pupils after the data cleaning procedure, described in Section 3.1. Participants were split into a STEM and no-STEM group based on their answer about a preferred future profession. People who preferred engineering, IT, economics/accounting, science, healthcare were assigned to the STEM group. In total, 332 participants were assigned to the STEM group (144 female), 217 to the no-STEM group (130 female).

A full description of the sample by age, gender and STEM grouping is available in the Table 1.

### 2.2. Measures

#### 2.2.1. Cognitive Abilities

**Figure A1:**
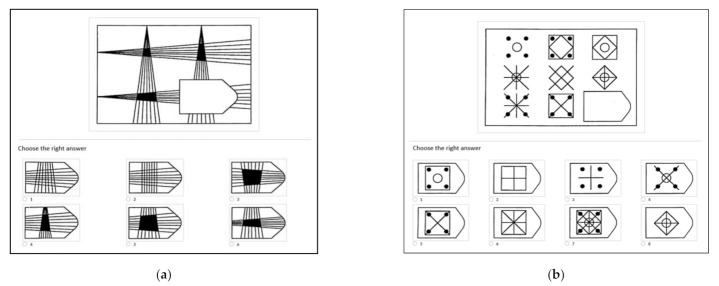
Example of the tasks Raven’s Standard Progressive Matrices (**a**) series A and (**b**) series F.

Raven’s Standard Progressive Matrices. One of the most “pure” measurements of the general intelligence factor g, highlighted by Spearman, is the Raven Standard Progressive Matrices test. The test is one of the best measures of non-verbal intelligence and is closer to tests of mathematical competence in terms of stimulus material and required mental operations (i.e., abstract figural and numerical material) [75]. This test is implemented for online testing [74]. Raven’s Standard Progressive Matrices consist of 60 tasks, which are grouped into 6 series (A, B, C, D, E, F). Each series consists of 12 matrices. The participant must select the missing element of the matrix among the options offered. The total time is 20 min. Examples of the tasks are presented in the Appendix A, Figure A1.

**Figure A2:**
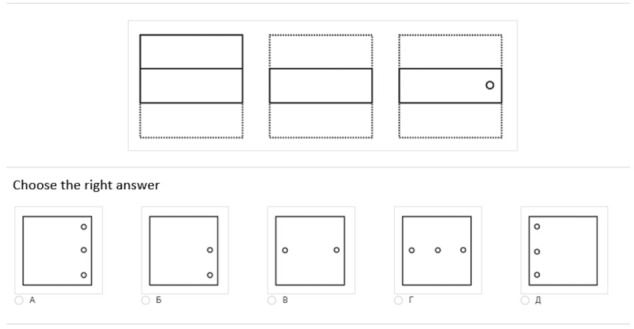
Example of tasks of “Paper folding” Test.

**Figure A3:**
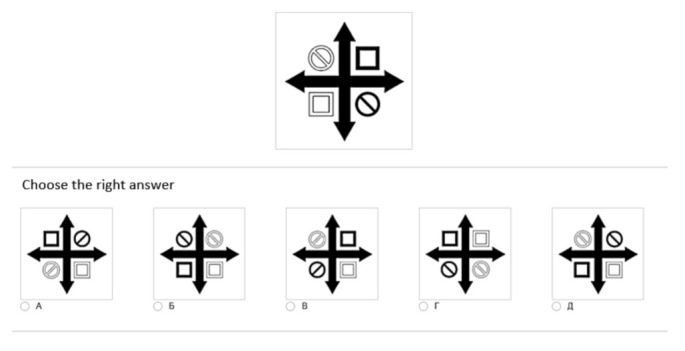
Example of tasks of “Shape rotation” Test.

**Figure A4:**
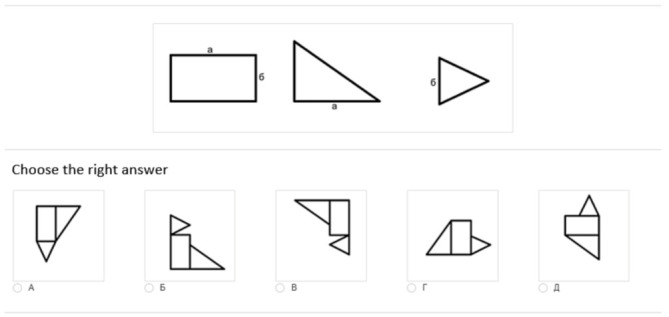
Example of tasks of “Pattern assembly” Test.

Spatial ability test battery. Spatial ability was tested by using tree subtests from original online gamified battery ‘King’s Challenge’ [76] adapted to Russian sample [77]. The battery includes tests for evaluation of spatial abilities such as spatial visualization (“Paper folding”), spatial rotation (“Shape rotation”), spatial relations (“Pattern assembly”). The total score on all three tests shows the level of spatial ability. Maximum score is equal to 30. For each task in the tests, a time limit of 20 s is given. The total battery administration time is 20 min. Cronbach’s alpha for subtests on Russian students ranged from 0.77 to 0.91. A detailed description of the test tasks in the battery are given below, examples of the tasks are in the Appendix A, Figure A2, Figure A3 and Figure A4.

“Paper folding” Test. Participants see a piece of paper folded in several steps. On the last image in each row there is a point denoting a through hole. Participants are asked to determine which of the 5 figures on the right shows how the holes will be located when the sheet of paper is fully unfolded. The test includes 15 tasks for spatial visualization.

“Shape rotation” Test. Participants are invited to determine which of the figures (answer options A–E) is identical to the sample, provided that the sample was turned clockwise. The test includes 15 tasks for spatial rotation.

“Pattern assembly” Test. Participants are asked to identify the form, which is composed of a set of polygonal components presented in the stimulus; these parts are marked with letters indicating where they should be connected. The test includes 15 tasks for spatial relationships.

Spatial ability score is the sum of the three scales: “Paper folding”, “Shape rotation”, “Pattern assembly”.

#### 2.2.2. Self-Perceived Ability and Intrinsic Value

Based on the self-report, this questionnaire assesses a student’s motivation in relation to different components of school education, as well as self-assessment of their own abilities in various fields, including issues related to basic school subjects [78].

For assessing self-perceived ability in mathematics, science, and academic achievements, students were asked to indicate on a 5-point Likert scale “how good they think they were” in various types of activities (for example, “How good do you think you count in your mind?”; “How good do you think you are at studying nature and living beings?”). Intrinsic motivation for mathematics, science, and academic achievements was assessed by asking questions on how much students liked particular tasks (for example, “How much you like to count in your mind?”; “How much you like studying nature and living beings?”). Total scores for all disciplines indicate overall intrinsic motivation for learning and self-perceptions of academic achievement.

#### 2.2.3. Career Preferences

Students completed a survey for a future career they would find preferable. Students were asked to choose the three most preferable professions in their opinion. According to the results, they were selected into two groups: the first group of schoolchildren who put a STEM profession in the top of their preferred careers (for example, a doctor, programmer) and the second group of schoolchildren who preferred professions not related to the direction of STEM (for example, journalist, psychologist).

#### 2.2.4. Gender Stereotypes and Incremental Beliefs about STEM

We developed an additional questionnaire with 4 scales in order to measure the background variables in our study: perceived difficulty of math (4 items, e.g., “Math is harder for me than other subjects”), STEM-related gender stereotypes (4 items, e.g., “In general, girls are less interested in STEM than boys”), educational aspirations in math (6 items, e.g., “I should study math, because it will help me find a job”) and friends’ attitudes towards math (3 items, e.g., “Most of my friends are good at math.”). Participants scored all items on the same 4-point Likert scale (with 2 negative and 2 positive ratings). Time was a constraint in this study, so our aim with these questionnaires was to make them as short as possible while retaining acceptable reliability. Cronbach’s alpha reliability for these questionnaires was at or above 0.74. Translated versions of these additional scales and their individual reliability coefficients are available in the Appendix A, Table A1. Instead of the raw scores, we chose to use factor scores for this questionnaire. In order to obtain them, we applied PCA with oblimin rotation and 4 components. The number of components was initially chosen based on the expected structure of the STEM questionnaire (we expected 4 distinct scales), and it was also supported by parallel analysis.

### 2.3. Data Analysis

#### 2.3.1. Descriptive Statistics and ANCOVA

Analysis was performed in R 4.0.3. Descriptive statistics were used to provide an overview of the data. Network analysis was performed in R (https://www.R-project.org/, accessed on 9 February 2022) using the “bootnet” [79], “igraph” [80] and “qraph” [81] packages. For the non-parametric transformation, the “huge” package was used.

In order to compare groups by STEM preference and gender, we had to control for the effects of age, so we performed an ANCOVA with type III SS and post hoc Tukey HSD using age as a covariate. To make the results more interpretable, we centered the age variable for this analysis (i.e., subtracted the sample average from each participant’s age).

#### 2.3.2. Network Analysis

In the present study, the network analysis was used to examine the structure of connections among cognitive functions, intrinsic motivation and incremental beliefs; the Gaussian Graphical Model Networks (GGM) were used to examine the structure in the traits set [79]. The network is constructed based on a partial correlation matrix with a graphical lasso regularization, and the absence of connection between nodes indicates the conditioning of this connection by other nodes. The network analysis is more informative than correlational analysis [82]. Prior to the network estimation, the data were transformed via huge.npn function from huge package to relax the constraint of the normal distribution. For negative values in the adjacency matrix, their absolute value was used.

To evaluate the structure of the network, several measures were used. The centrality measures characterize the importance of the node in the network. Betweenness centrality of a node is the number of the shortest paths between any two nodes that pass through this node. The shortest path is a “distance” between two nodes that indicates the ease of transition between nodes. The shortest paths pass through the node, the more central the node is, thus, the more important it is. Closeness centrality is the inverse of the sum of all shortest paths. In the case of the closeness centrality, the central nodes have many strong connections to the other nodes and, thus, are closer in distance to the other nodes, thus, more important in the network. Another important parameter is the Degree—the sum of the node connection weights. In the case of the weighted graph, the degree represents the number and the strength of the connections. We use the degree as well to represent connection strength. Nodes with higher degree have more connections that are stronger and could thus be considered more important in the network. Clustering measures allow investigating the clusters of nodes appearing in the network. A cluster of nodes is defined as a group of nodes, with more connections between the nodes within the cluster than connections with the nodes outside the cluster. The main idea in the clustering coefficient is based on the detection of triangles in which the nodes participate. Each network was also divided into a set of clusters via the Clique Percolation Method [83].

The Clique Percolation Method is a novel graph-theoretic technique that allows detection of overlapping communities (clusters) within the network. The CPM is important in the psychological traits’ analysis as it is possible to have a trait that is strongly linked with different communities. We consider two communities overlapping if there is at least one node that belongs to both communities. For better selection of important nodes and connections from each network, the minimum spanning tree (MST) was extracted. The minimum spanning tree is a subset of edges that connects all vertices with the minimum possible total weight sum, the skeleton of the network. The minimum spanning tree gives an insight into the strongest edges in the network that array the most essential information. The MST’s also reflect the importance of the nodes and groupings in the network.

## 3. Results

In this section, we present descriptive statistics of our sample and the group differences in cognitive abilities, intrinsic motivation and ability self-perceptions in math and science and also gender stereotypes and incremental beliefs about STEM in the STEM and non-STEM groups and for boys and girls. We then discuss the network structure of psychological characteristics in these groups.

### 3.1. Descriptive Statistics for Sample

We removed all cases with over 10 missing responses, leaving 615 total participants in the study. Note that the perceived difficulty of math scale, which can be considered as reverse-scored (since a higher score means more difficulties), has been reversed. Further, Mahalanobis distance was used to deal with the multivariate outliers. Quantile 0.9 of the Mahalanobis distance was used to eliminate the outliers. After controlling for outliers, 546 participants remained for the analysis. A table with descriptive statistics is presented in the Appendix A, Table A2.

### 3.2. ANCOVA Test Results for Groups

In this section, we present the main results of ANCOVA in cognitive abilities, intrinsic motivation and self-perception and incremental beliefs. The full results are in the Appendix A, Table A3.

#### 3.2.1. Cognitive Abilities

**Figure 1:**
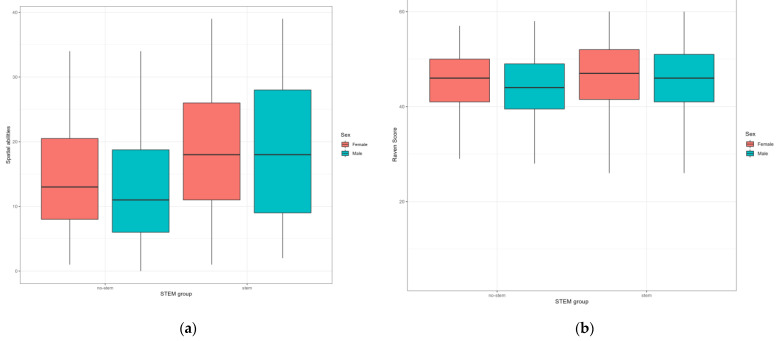
Group values of spatial abilities (**a**) and Raven score (**b**) in STEM and No-STEM groups.

In this section, we summarize the differences we observed in adolescents’ cognitive abilities. Figure 1 shows the differences in non-verbal intelligence and spatial abilities for adolescents in the STEM and No-STEM groups, divided by sex.

We can observe significant differences in non-verbal intelligence (F = 4.645; *p* < 0.034 ŋ^2^ = 0.011) and spatial abilities (F = 19.842; *p* < 0.001; ŋ^2^ = 0.051) between the STEM and No-STEM groups. Adolescents in the STEM group have higher scores on all the scales under consideration. We observed no significant differences based on sex. Additionally, there was no interaction between sex and the STEM group.

#### 3.2.2. Intrinsic Motivation and Ability Self-Perceptions in Math and Science

**Figure 2:**
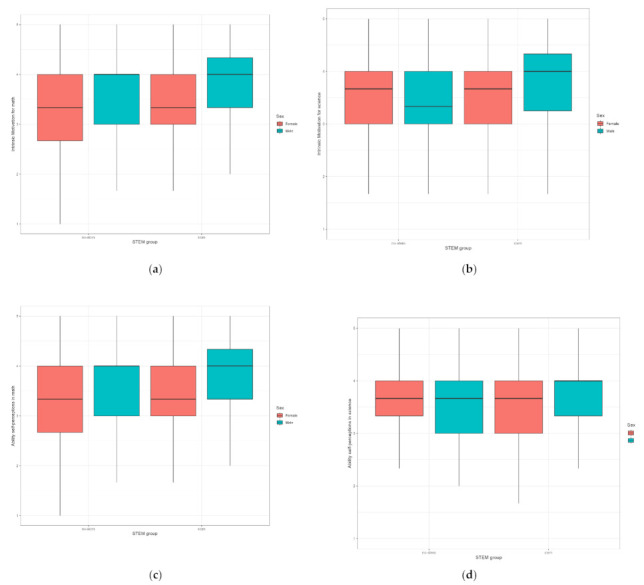
Group values for intrinsic motivation in math (**a**), ability self-perception in math (**b**), intrinsic motivation in science (**c**) and ability self-perception in science (**d**).

In this section, we review the differences in adolescents’ intrinsic motivation and ability self-perceptions in math and science. Descriptive statistics for all variables under consideration are reported in Table A4 in the Appendix A. The resulting differences among the groups are shown in the Figure 2

Here, boys have scored significantly higher than girls on the following scales: academic ability self-perceptions (F = 4.129; *p* < 0.043; ŋ^2^ = 0.008), intrinsic motivation (F = 6.831; *p* < 0.009; ŋ^2^ = 0.012), intrinsic motivation for math (F = 14.881; *p* < 0.001; ŋ^2^ = 0.027) and ability self-perception in math (F = 5.684; *p* < 0.017; ŋ^2^ = 0.01). Once again, there were significant differences between the STEM and No-STEM groups. The STEM group scored significantly higher on intrinsic motivation for math (F = 4.702; *p* < 0.031; ŋ^2^ = 0.009), science (F = 5.059; *p* < 0.025; ŋ^2^ = 0.009) and ability self-perceptions in math (F = 3.896; *p* < 0.049; ŋ^2^ = 0.007) scales.

**Figure 3:**
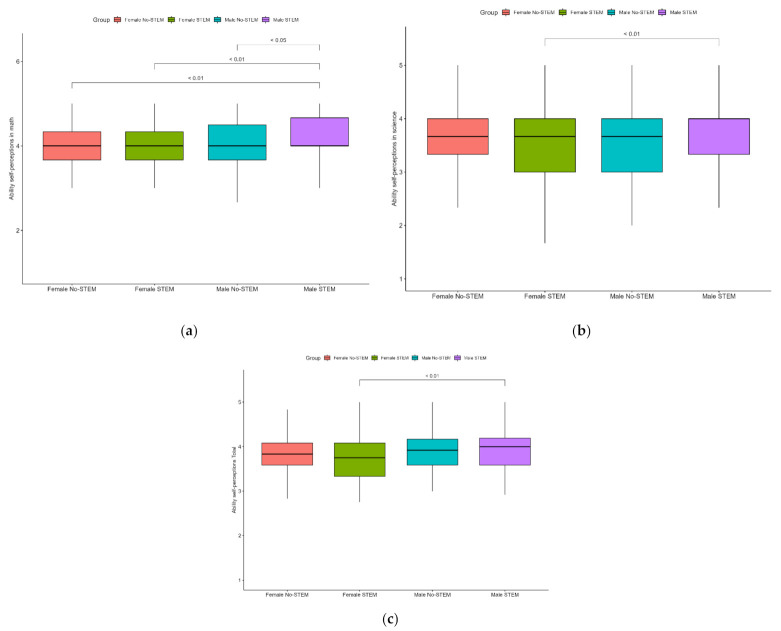
Post hoc analysis of ability self-perception in math (**a**), science (**b**) and in total (**c**) by sex and STEM/No-STEM groups.

We also investigated the interactions between groups by STEM and sex in relation to the scales in our study. For the statistically significant differences, means are presented in the Figure 3, and a table with all results of this analysis is available in the Appendix A (see Table A5). Boys from the STEM group were found to have significantly higher scores on the ability self-perceptions in math scale than all the other groups: boys from the No-STEM group (diff = 0.23, *p* = 0.03), girls from the STEM group (diff = 0.24, *p* > 0.01) and girls from the No-STEM group (diff = 0.24, *p* > 0.01). Finally, within the STEM group, boys had significantly higher scores than girls on the following scales: ability self-perceptions in science (diff = 0.21, *p* = 0.04) and self-perception of academic ability (diff = 0.18, *p* > 0.01).

#### 3.2.3. Gender Stereotypes and Incremental Beliefs about STEM

**Figure 4:**
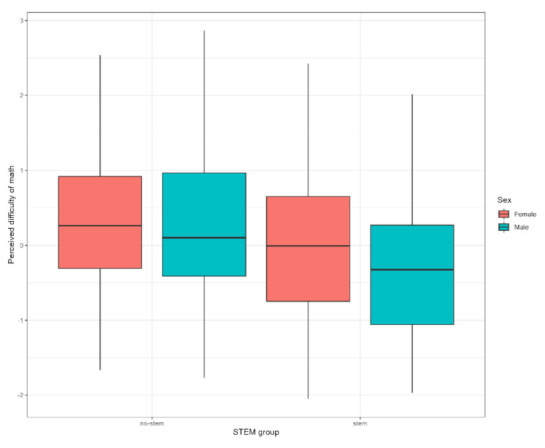
Group values in perceived difficulty in math in STEM and No-STEM groups.

According to the results of the comparison of groups by sex, boys scored significantly higher on the educational aspirations in math (F = 5.677; *p* < 0.018; ŋ^2^ = 0.01) and the STEM-related gender stereotype (F = 41.373; *p* < 0.001; ŋ^2^ = 0.053) scales. Predictably, they also scored significantly lower on the perceived difficulty of math (F = 5.725; *p* < 0.017; ŋ^2^ = 0.01) scale, which is reversed (see Figure 4).

**Figure 5:**
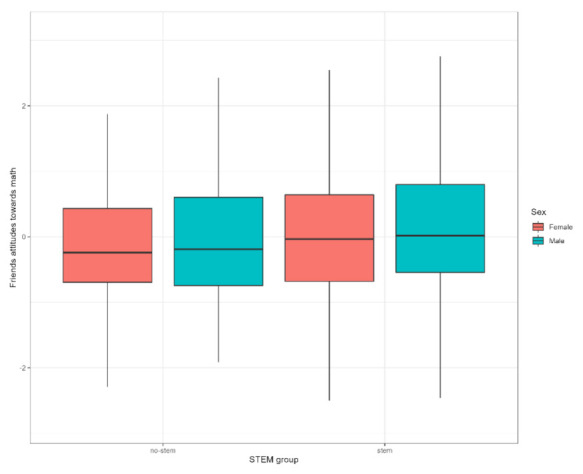
Group values in friends’ attitude towards math in STEM and No-STEM groups.

Boys perceive math as being easier, while girls think that math is less important for their future. Girls also think that other girls are not very interested in or good at STEM subjects. Adolescents in the STEM group scored lower on the perceived difficulty of math (F = 27.133; *p* < 0.001; ŋ^2^ = 0.048) scale. They also view math as more important for future careers (F = 6.356; *p* < 0.012; ŋ^2^ = 0.012) than their counterparts from the No-STEM group. We observed no differences by sex or STEM group on the friends’ attitudes towards math scale, and no interaction effects within groups were detected either (see Figure 5).

### 3.3. Network Analysis Results for Groups

For this part of the study we have constructed four networks, one for each group: STEM, No-STEM, boys and girls. Network parameters were estimated via GGM with EBICglasso regularization. The graphs were visualized with qgraph, and node color is used to denote clusters. Statistics for every node in the networks are available in the Appendix A, Table A5.

#### 3.3.1. STEM and No-STEM Group Networks

**Figure 6:**
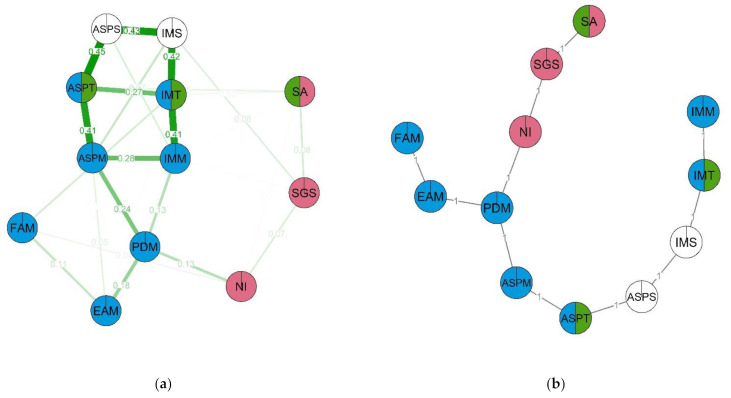
Estimated network (**a**) and corresponding MST (**b**) for the STEM group. Thicker lines between variables indicates stronger connection; different colors indicate membership of variables to a specific cluster. Two-color nodes belong to two clusters simultaneously. Three clusters emerge in the network: (1) NI—Non-verbal intelligence, SA—Spatial ability, SGS—STEM-related gender stereotypes; (2) SA—Spatial ability, ASPT—Ability self-perceptions, IMT—Intrinsic motivation; (3) ASPT—Ability self-perceptions, IMT—Intrinsic motivation, ASPM—Ability self-perceptions in math, EAM—Educational aspirations in math, IMM—Intrinsic Motivation for math, FAM—Friends’ attitudes towards math, PDM—Perceived difficulty of math. Non-clustered: ASPT—Ability self-perceptions.

In the STEM group network (Figure 6), the following nodes had the highest levels of centrality: academic ability self-perception (ASPT), ability self-perception in science (ASPS) and intrinsic motivation for science (IMS). This network contained three clusters. The first cluster (red) included non-verbal intelligence, spatial abilities and STEM-related gender stereotypes. The second cluster (green) consisted of spatial abilities, academic ability self-perception and intrinsic learning motivation. The third cluster (blue) included ability self-perception in math, importance of math for future career, friends’ attitudes towards math and intrinsic motivation for learning math. The strongest group of nodes (with the highest node strength) in this network is made up of perceived math difficulty, math self-efficacy and the importance of math for future career (PDM, ASPM and SGS). Notably, gender stereotypes are located in the same cluster as spatial abilities. Overall, we can observe from the network graph that spatial abilities are related to self-perception of one’s skills and motivation both directly and indirectly through perceived difficulty of math (which, in turn, is related to educational aspirations in the subject and friends’ attitude towards it). Some of the nodes did not create notable clusters: non-verbal intelligence, academic self-efficacy and intrinsic motivation for science (NI, IMS, ASPS).

**Figure 7:**
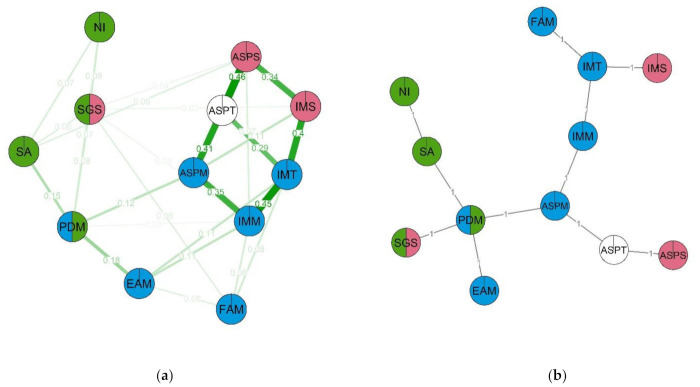
Estimated network (**a**) and corresponding MST (**b**) for the No-STEM group. Thicker lines between variables indicates stronger connection; different colors indicate membership of variables to a specific cluster. Two-color nodes belong to two clusters simultaneously. Three clusters emerge in the network: (1) NI—Non-verbal intelligence, SA—Spatial ability, SGS—STEM-related gender stereotypes, PDM—Perceived difficulty of math; (2) SGS—STEM-related gender stereotypes, ASPS—Ability self-perceptions in science; IMS—Intrinsic Motivation for science; (3) PDM—Perceived difficulty of math, EAM—Educational aspirations in math, IMM—Intrinsic Motivation for math, FAM—Friends’ attitudes towards math, ASPM—Ability self-perceptions in math, IMT—Intrinsic motivation. Non-clustered: ASPS—Ability self-perceptions in science; IMS—Intrinsic Motivation for science.

In the No-STEM group network (Figure 7), the most important nodes appear to be academic self-efficacy, specifically, self-efficacy in science, as well as intrinsic motivation towards learning (ASPT, ASPS, IMT). However, in this case, node centrality is lower, which informs us that this is a more sparse network. As with the previous network, we can observe three clusters. The first cluster (red) contains non-verbal intelligence, spatial abilities, math ability self-perception and STEM-related gender stereotypes. The second cluster (red) is made up of science ability self-perception, intrinsic motivation for science and STEM-related gender stereotypes. The third cluster (blue) contains intrinsic motivation for math, math ability self-perception, importance of math for future career and perceived difficulty of math. The structure of this network is similar to that previous; however, in the STEM group network, math-related self-efficacy and intrinsic motivation were linked via spatial abilities, while in the No-STEM group network, the linking node was instead STEM-related gender stereotypes. As well as that, a notable connection emerges between STEM-related gender stereotypes and friends’ attitudes towards math. On the MST graph we can see that STEM-related gender stereotypes are linked with math self-efficacy and intrinsic motivation for math via the perceived difficulty of math node. The following nodes have the highest clustering coefficients in the No-STEM group network: non-verbal intelligence, spatial abilities, math importance for future career and friends’ attitudes towards math (NI, SA, EAM, FAM). The nodes with the lowest clustering coefficients are academic and math ability self-perception, as well as intrinsic learning motivation.

#### 3.3.2. Male and Female Networks

**Figure 8:**
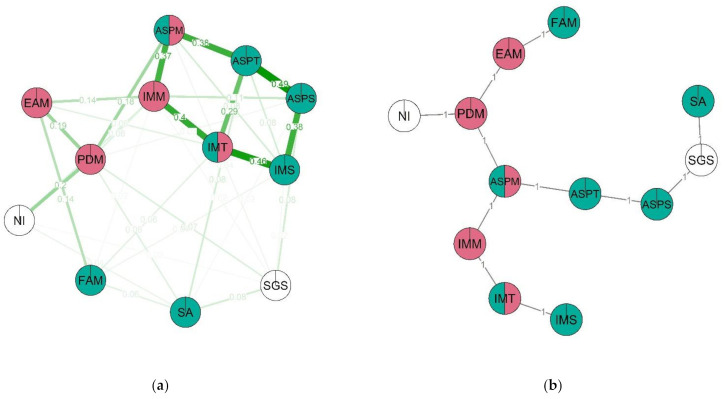
Estimated network (**a**) and corresponding MST (**b**) for the females. Thicker lines between variables indicate stronger connection; different colors indicate membership of variables to a specific cluster. Two-color nodes belong to two clusters simultaneously. Two clusters emerge in the network: (1) SA—Spatial ability, FAM—Friends’ attitudes towards math, IMS—Intrinsic Motivation for science, ASPS—Ability self-perceptions in science, ASPT—Ability self-perceptions, ASPM—Ability self-perceptions in math, IMT—Intrinsic motivation; (2) IMT—Intrinsic motivation, ASPM—Ability self-perceptions in math, IMM—Intrinsic Motivation for math, PDM—Perceived difficulty of math, EAM—Educational aspirations in math. Non-clustered: NI—Non-verbal intelligence, SGS—STEM-related gender stereotypes.

Now let us review the second pair of networks. This time participants were grouped by sex. In the girls’ network (Figure 8), the nodes with the most centrality are academic self-efficacy, math self-efficacy and intrinsic motivation for math (ASPM, IMM, ASPT). The most connected nodes (i.e., nodes with the highest clustering coefficients) are non-verbal intelligence, math importance for future career, STEM-related gender stereotypes and perceived difficulty of math (NI, EAM, SGS, PDM). In terms of MST (and centrality values), the most important node is self-efficacy in math. Three branches diverge from it, including a branch with intrinsic motivation, a branch with an aspiration of maths for students and their friends and the level of non-verbal intelligence, and also a branch with an assessment of abilities including self-efficacy in learning and science and also the level of spatial abilities. This network includes two clusters. The first cluster (green) contains spatial abilities, friends’ attitudes towards math, academic, math and science self-efficacy, intrinsic motivation for learning in general and for science. The second cluster (red) consists of math importance, subjective difficulty of math, intrinsic motivation for math, math self-efficacy and intrinsic motivation towards learning.

**Figure 9:**
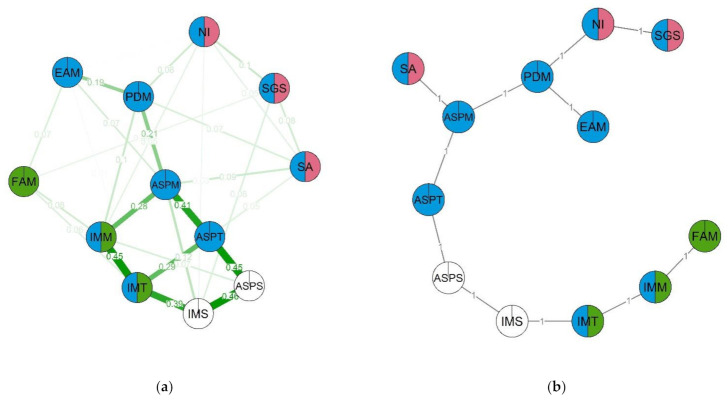
Estimated network (**a**) and corresponding MST (**b**) for the males. Thicker lines between variables indicate stronger connection; different colors indicate membership of variables to a specific cluster. Two-color nodes belong to two clusters simultaneously. Three clusters emerge in the network: (1) IMM—Intrinsic Motivation for math, PDM—Perceived difficulty of math, EAM—Educational aspirations in math, IMT—Intrinsic motivation, ASPT—Ability self-perceptions, ASPM—Ability self-perceptions in math, SA- Spatial ability; NI—Non-verbal intelligence, SGS—STEM-related gender stereotypes; (2) SA—Spatial ability; NI—Non-verbal intelligence, SGS—STEM-related gender stereotypes; (3) FAM—Friends’ attitudes towards math, IMT—Intrinsic motivation; IMM—Intrinsic Motivation for math. Non-clustered: ASPS—Ability self-perceptions in science; IMS—Intrinsic Motivation for science.

In the boys’ network (Figure 9), the nodes with the highest centrality coefficients are science and academic self-efficacy and intrinsic motivation for science (IMS, ASPS, ASPT). The overall lower clustering coefficients in this network are lower; it is more sparse than the girls’ network. The most clustered nodes are perceived difficulty of math and academic self-efficacy (PDM, ASPT). As with the other networks, three node clusters emerge; however, in this case, one cluster dominates the other two. The first and largest cluster (blue) contains perceived math difficulty, math importance for future career, intrinsic motivation towards learning in general and math specifically, academic and math self-efficacy, non-verbal intelligence and spatial abilities. The second cluster (red) consists of non-verbal intelligence, spatial abilities and STEM-related gender stereotypes. The third cluster (green) contains friends’ attitudes towards math, intrinsic motivation for math and for learning in general. The MST structure of this network suggests that it splits into two blocks: motivational (consisting of friends’ attitudes towards math and intrinsic motivation for math, science and learning in general) and cognitive (spatial abilities and self-efficacy nodes).

Additionally, our network analysis demonstrates that STEM-related gender stereotypes are less impactful in the boys’ network: the degree of this node is 4, compared to 7 in the girls’ network, node power is 0.28 compared to 0.32 and the clustering coefficient is higher (0.18 compared to 0.17, respectively). This is also demonstrated by the lower weights of the edges connected to this node in the boys’ network.

## 4. Discussion

### 4.1. Group Differences in STEM-Related Chacteristics

One objective of our study was to discover what is different about adolescents who choose STEM career paths. We hypothesized that adolescents who prefer STEM would differ in their cognitive skills and their disposition towards STEM disciplines. As expected, we observed significant differences between the two groups in our study: students who chose STEM have higher non-verbal intelligence and spatial abilities. They have significantly higher intrinsic motivation towards learning math and science, they are more confident in their math skills and see math as being easier than do their counterparts who did not choose STEM. Perhaps as interesting were the areas where we did not find significant differences. As such, the groups did not differ on their overall motivation towards learning, their overall academic skills or STEM-related gender stereotypes (for example, “girls are less interested in STEM”, “girls need to be more like boys to succeed in STEM”, etc.).

These results fall in line with previous findings observed on contrasting samples of average and gifted participants that spatial abilities are a reliable predictor of later achievements in STEM areas [10,84]. One of these studies conducted by Wai et al. [7] demonstrated that students who were interested in STEM, later earning a bachelor’s or master’s degree in a STEM area, had higher spatial abilities at 13 years old. In a recent study by Moe et al. [66], university students from STEM specialities were shown to have higher scores on the mental rotation task than other students (5% effect size). In another study, Wang [3] approached the same topic within the framework of social cognitive career theory [5], finding that students’ choice to go into STEM had been influenced by their performance in math and natural science subjects in middle school, their familiarity with the same subjects, their self-efficacy in math and their future career plans.

Based on the research available on the topic of sex differences in STEM, we expected that boys in general would score higher on variables related to cognition, such as non-verbal intelligence and spatial abilities, while girls would have more pronounced STEM-related gender stereotypes. This has been partially confirmed by our findings. First, the boys group in our study did score significantly higher on science, math and overall academic self-efficacy, intrinsic learning motivation and math importance for future careers. Some previous research also concluded that girls undervalue their math skills in comparison to boys, even within the same level of math achievement [50,51,52,53]. This is true for both primary school students (for example [54]) as well as for secondary to high school students (for example [55]). Girls in general are also less interested in math and have lower instrumental motivation towards it [25,56,57]. Boys are more oriented towards achievement in math-related areas and choose math more often for further study [45,46,58].

That said, we did not observe significant differences in cognitive abilities between the sexes: girls and boys in general did not differ in their spatial abilities, or in non-verbal intelligence. This is a departure from previous findings, which indicate that there are significant differences between the sexes in terms of spatial abilities [49,85]. In a study of sex differences in mental rotation tasks, Geiser [86] reported that boys did better than girls over the whole age range from 9 to 23 years old. At the same time, in recent years, studies have noted a reduction in differences in cognitive characteristics in men and women (gender-similarity hypothesis) [87,88]. It is also noted that the difference in spatial abilities, spatial visualization in particular, is secondary for girls choosing STEM [89]. Thus, perhaps in this we are observing a common trend towards higher sex parity in STEM.

One more study objective was to investigate the gender gap in STEM, particularly regarding STEM-related stereotypes. As expected, girls scored higher on the gender stereotypes scale, which in our study included notions such as “girls are in general less interested in STEM” and “girls need to be more like boys to succeed in STEM”. Girls also displayed lower self-efficacy, even though there were no significant differences in objectively measured spatial abilities and non-verbal intelligence compared to boys. Given that we did not find differences in the cognitive sphere between boys and girls, gender stereotypes are highlighted as a possible reason for sex disparity in STEM areas (e.g., [48,49]. Thus, women could have lower math self-efficacy than men with the same level of math achievement, which can influence women to leave STEM [90].

### 4.2. The Network Structure and the Role of Gender Stereotypes

To investigate how gender-related STEM stereotypes, cognitive abilities, self-perceived ability and intrinsic value are related to the sex and career preference (STEM or No-STEM) in school students we split our sample into four groups (male STEM, female STEM, male No-STEM, female No-STEM) and made pairwise comparisons. We observed significant differences in math, science and overall academic self-efficacy between these groups (note that unlike some of the researchers in this area, we did not correct for actual achievement). Boys from the STEM group have the highest science and academic self-efficacy, followed by girls from the STEM group and then boys from the No-STEM group. Larson et al. [91] propose that math and science self-efficacy in STEM students is a strong predictor of follow-through (earning a bachelor’s degree, for example). In their study, math and science self-efficacy were a predictor of graduating from university as long as 4–8 years after the initial measurement. Factoring in these self-efficacy variables also improved prediction accuracy of the chance of the participants dropping out before graduation by 4.4%. This may be one of the explanations why more men pursue a STEM career than women.

As well as individual and gender-related differences, we were also interested in identifying whether STEM-related stereotypes have a special place among other characteristics in the groups by sex and STEM preferences. The network analysis of the structure of the psychological characteristics in the STEM and non-STEM groups shows that for both groups there is similarity in the two main groups of variables: “the cognitive abilities” and “stem attitudes”, but each group has specificity in the relation between the two factors. In the STEM group, the spatial abilities seem to have a substantial effect on the attitude towards STEM as it is connected with selfperceived ability and motivation in learning in the separate cluster outside the other two. Perceived difficulty of math seems to be important as it is central in the MST graphs and has strong connections between both clusters. In the non-STEM group, the structure of connections, mostly similar to the STEM group, has some unique features. First, the STEM-related gender stereotypes separate into the cluster with self-perception of ability and motivation in science and have a strong connection with perceived difficulty of math. The latter is also strongly associated with the cognitive cluster via the spatial abilities, while in the STEM network, it is connected via non-verbal intelligence. The cognitive cluster in the No-STEM group is also more interconnected than in the STEM group. Overall, the STEM-related gender stereotypes in both groups seem to be connected with cognitive abilities, but it is more strongly associated with them in the No-STEM group. The main differences between the structures of the groups were that in the STEM group the self-perceived ability in math played a higher role, while in the No-STEM group intrinsic motivation to study math was more important.

In the female group, cluster structure is quite different. The separate cognitive cluster is missing, and the psychological traits are separated into two clusters, “motivation to math” (motivation, aspiration, perceived difficulty of math and intrinsic motivation) and “cognitive abilities and ability self-perceptions” (spatial abilities, abilities self-perception overall and in math and science, also with interest and motivation to study math in friends). The STEM-related gender stereotypes trait does not fall into a separate cluster, but it has a wide set of connections with other traits. On the MST graph the first cluster seems to play a key role in the structure, which might indicate that females are more prone to the motivation traits (including self attitude and influence from the environment). The STEM-related gender stereotypes’ influence is characterised as quite wide but not very strong.

In the male group, one cluster integrating almost all traits is defined. As subclusters, the cognitive characteristics and combination of intrinsic motivation, motivation to math and friend motivation to study math are distinguished. This cluster might indicate the importance of friends and intrinsic motivation. Overall, in the male group, STEM-related gender stereotypes are connected with the cognitive characteristics, while in the female group the STEM-related gender stereotypes effect is spread across the whole trait set. In addition, motivational traits are different in the two groups. In the male group there is one cluster of their’s and friends’ motivation to study math, aspiration and difficulty for math with self-perception of abilities in it and intrinsic motivation traits, and in the females it is divided into two—cognitive abilities and ability self-perception traits and aspiration and difficulty in math with intrinsic motivation. In the female group, ability self-perception is linked with friends’ attitude more than in the male group and is linked specifically with spatial abilities.

Taken as a whole, in the network analysis structures of psychological characteristics, the most central factors are the self-perception of academic achievements and intrinsic motivation both for boys and for girls. At the same time, while for boys these factors are directly associated with the level of objective (spatial) abilities, for girls the effect is indirect: the objective levels of cognitive performance affected the self-perception of academic achievements and intrinsic motivation through the level of gender stereotypes, perceived difficulty of math, self-perception of abilities in science and other factors. We hypothesize that the more complicated structure of the relationship between the objective level of abilities and the most important factors for choosing a STEM career in girls may be one of the reasons for the well-established STEM gender gap. For the boys “the road to STEM” appears to be more direct: if a boy has the abilities that “fit” STEM demands, he is more likely to choose STEM professions as a future career. For girls, having the abilities is not enough: there are multiple stereotype-related factors that reduce the chances of them seeing their future in STEM.

## 5. Conclusions

Thus, in our study, it was shown that for girls, gender stereotypes, together with the general self-esteem of their abilities, is associated with the formation of self-esteem of abilities in the field of mathematics, one of the important factors that distinguishes groups of adolescents who choose and do not choose a STEM discipline as a future career. Despite the fact that gender stereotypes were associated with self-esteem of abilities not only in girls but also in boys, the contribution of this factor to the formation of the general system of psychological characteristics in boys was significantly lower, as evidenced by both the low values of the centrality of this variable and low absolute values of the weights of the links of this variable in the network. Despite the variety of factors influencing the choice of STEM in adolescence, we can say that gender stereotypes associated with STEM areas are one of the central barriers for women to enter STEM. One of the solutions to reduce the negative consequences of stereotypes in this area may be teaching gender diversity, which is lacking in Russia. Existing research suggests that diversity of education that aims to learn through stereotyping [92,93] and raising awareness of implicit bias [94], can lead to more positive attitudes towards reference groups. When women identify with science and the STEM direction is part of their self-identity, they see their perspectives in developing themselves in this area. The goal of future research is to investigate the dynamic changes in factors and barriers that prevent entry and the pursuit of a career in STEM, as well as track the effect of diversity education on existing stereotypes.

## 6. Limitation

There are several limitations in our research. First, STEM-related gender stereotypes are measured with a questionnaire that already assumes a certain stereotypical mindset, and we only inquire about the extent to which our respondents agree with this specific set of stereotypes. We know from the literature review that even phrasing questions in such a way can impact reporting, so this is a limitation we should keep in mind when expanding upon this research. Second, in the network analysis, all connections were treated as positive; therefore, we cannot trace negative connections between our variables. Moreover, we did not set the task of comparing the structure of subgroups by sex and belonging in STEM; at the same time, we received results that indicate that boys from the STEM group differ from all other subgroups (STEM girls, non-STEM girls, non-STEM boys) in self-assessment of their abilities, etc. This leaves the question of whether the structure of this group should be treated separately. The reason for the identified differences may be due to the fact that it is the specific characteristics of that group that allow them to pursue STEM. This question requires further investigation. We also cannot argue that adolescents who prefer the STEM direction in our study will actually choose it in the future, we can only talk about prerequisites that can facilitate and resist the entrance to STEM. Third, in the present study, we did not directly assess the family and teacher effects on gender stereotypes. The influence of the family and school environment is still to be studied in the future.

## Figures and Tables

**Table 1:** Sample size by age for sex and preferences in STEM, no-STEM groups.

	Sex (N)			STEM (N)			No-STEM (N)		
Age	All	Boys	Girls	All	Boys	Girls	All	Boys	Girls
12	24	12	12	18	10	8	6	2	4
13	100	50	50	65	38	27	35	12	23
14	151	81	70	91	52	39	60	29	31
15	96	49	47	54	32	22	42	17	25
16	143	67	76	83	46	37	60	21	39
17	32	14	18	20	10	10	12	4	8
Total	546	273	273	331	188	143	215	85	130

## Data Availability

The datasets generated and analyzed during the current study are available from the corresponding author on reasonable request. None of the experiments were preregistered.

## Appendix A

**Table A1:** Questionnaire “Gender stereotypes and incremental beliefs about STEM”.

Variable Name (Code)	Number of Items	Chronbach’s Alpha	Items
Perceived difficulty of math	4	0.8	I usually do well in math (reverse-coded). Math is harder for me than for many of my classmates. Studying math gives me anxiety. Math is harder for me than other subjects.
STEM-related gender stereotypes	4	0.74	Overall, girls are less interested in a STEM career than boys. Girls usually have less knowledge and skills that are necessary for STEM disciplines. In order to succeed in STEM, girls need to be more like boys. Teachers usually support boys’ interest in STEM more than they do for girls.
Educational aspirations in math	6	0.75	I must study math since it will help me get a job. If I try hard enough, I can succeed in math. My success in math is due to myself and nobody else. If I wanted, I could be good at math. My parents think that… … studying math is interesting. … studying math is important for a future career.
Friends’ attitudes towards math	3	0.76	Most of my friends… … are good at math. … study math hard. … are interested in math.

**Table A2:** Descriptive statistics.

	All		Boys		Girls		STEM		No-STEM	
	N	M(SD)	N	M(SD)	N	M(SD)	N	M(SD)	N	M(SD)
Non-verbal intelligence	414	44.6 (9.09)	211	44 (9.59)	203	45.2 (8.51)	248	45.2 (8.9)	166	43.6 (9.31)
Spatial ability	369	16.7 (9.75)	185	16.5 (10.4)	183	16.7 (9.04)	216	18.5 (9.98)	153	14.1 (8.82)
Self-perception of academic ability	546	3.84 (0.53)	273	3.89 (0.55)	273	3.79 (0.5)	331	3.83 (0.53)	215	3.86 (0.54)
Intrinsic values (motivation)	546	3.61 (9.62)	273	3.68 (0.62)	273	3.54 (0.6)	331	3.62 (0.6)	215	3.59 (0.64)
Intrinsic motivation for math	546	3.6 (0.86)	273	3.76 (0.82)	273	3.44 (0.88)	331	3.69 (0.8)	215	3.46 (0.94)
Ability self-perceptions in math	546	3.61 (0.85)	273	4.14 (0.65)	273	3.98 (0.62)	331	4.11 (0.6)	215	3.98 (0.67)
Ability self-perceptions in science	546	4.06 (0.64)	273	3.73 (0.75)	273	3.63 (0.7)	331	3.69 (0.74)	215	3.65 (0.7)
Intrinsic motivation for science	546	3.67 (0.73)	273	3.66 (0.84)	273	3.55 (0.86)	331	3.67 (0.85)	215	3.5 (0.84)
Perceived difficulty of math	546	2.40 (0.47)	273	2.31 (0.47)	273	2.49 (0.45)	273	2.51 (0.45)	273	2.33 (0.47)
STEM-related gender stereotype	546	2.02 (0.63)	273	2.18 (0.65)	273	1.87 (0.57)	331	2.02 (0.63)	215	2.02 (0.63)
Educational aspirations in math	546	2.96 (0.43)	273	3 (0.45)	273	2.93 (0.40)	331	2.89 (0.45)	215	3.01 (0.40)
Friends’ attitudes towards math	546	2.44 (0.58)	273	2.46 (0.60)	273	2.41 (0.57)	331	2.40 (0.58)	215	2.46 (0.58)

**Table A3:** ANCOVA Results.

		Sum Sq	Df	F Value	Pr (>F)	Levene Test	Eta2 Partial
Non-verbal intelligence	Int	773,446.4	1	9619.711	0	0.623	NA
	Age	574.063	1	7.14	0.008	0.623	0.017
	Sex	157.616	1	1.96	0.162	0.623	0.005
	STEM	373.445	1	4.645	0.032	0.623	0.011
	Sex–STEM	0.949	1	0.012	0.914	0.623	0
	Residuals	33,125.72	412	NA	NA	0.623	NA
Spatial ability	Int	88,870.01	1	982.838	0	0.007	NA
	Age	223.722	1	2.474	0.117	0.007	0.007
	Sex	60.356	1	0.667	0.414	0.007	0.002
	STEM	1794.16	1	19.842	0	0.007	0.051
	Sex–STEM	9.873	1	0.109	0.741	0.007	0
	Residuals	33,094.38	366	NA	NA	0.007	NA
Self-perception of academic ability	Int	7497.614	1	27,090.49	0	0.412	NA
	Age	0.963	1	3.481	0.063	0.412	0.006
	Sex	1.143	1	4.129	0.043	0.412	0.008
	STEM	0.148	1	0.534	0.465	0.412	0.001
	Sex–STEM	1.119	1	4.044	0.045	0.412	0.007
	Residuals	150.558	544	NA	NA	0.412	NA
Intrinsic values (motivation)	Int	6608.46	1	17,653.65	0	0.953	NA
	Age	0.676	1	1.805	0.18	0.953	0.003
	Sex	2.557	1	6.831	0.009	0.953	0.012
	STEM	0.008	1	0.02	0.887	0.953	0
	Sex–STEM	0.314	1	0.84	0.36	0.953	0.002
	Residuals	203.641	544	NA	NA	0.953	NA
Intrinsic motivation for math	Int	6522.011	1	9102.192	0	0.006	NA
	Age	0.294	1	0.411	0.522	0.006	0.001
	Sex	10.663	1	14.881	0	0.006	0.027
	STEM	3.369	1	4.702	0.031	0.006	0.009
	Sex–STEM	0.021	1	0.029	0.865	0.006	0
	Residuals	389.793	544	NA	NA	0.006	NA
Intrinsic motivation for science	Int	6522.374	1	9095.095	0	0.895	NA
	Age	0.597	1	0.832	0.362	0.895	0.002
	Sex	0.549	1	0.765	0.382	0.895	0.001
	STEM	3.628	1	5.059	0.025	0.895	0.009
	Sex–STEM	1.22	1	1.702	0.193	0.895	0.003
	Residuals	390.119	544	NA	NA	0.895	NA
Ability self-perceptions in math	Int	8312.319	1	20,918.59	0	0.365	NA
	Age	0.253	1	0.637	0.425	0.365	0.001
	Sex	2.259	1	5.684	0.017	0.365	0.01
	STEM	1.548	1	3.896	0.049	0.365	0.007
	Sex–STEM	1.364	1	3.434	0.064	0.365	0.006
	Residuals	216.167	544	NA	NA	0.365	NA
Ability self-perceptions in science	Int	6803.189	1	13,052.64	0	0.775	NA
	Age	1.42	1	2.725	0.099	0.775	0.005
	Sex	0.483	1	0.927	0.336	0.775	0.002
	STEM	0.221	1	0.424	0.515	0.775	0.001
	Sex–STEM	3.031	1	5.816	0.016	0.775	0.011
	Residuals	283.539	544	NA	NA	0.775	NA
Perceived difficulty of math	Int	1.603	1	1.732	0.189	0.275	NA
	Age	4.082	1	4.409	0.036	0.275	0.008
	Sex	5.3	1	5.725	0.017	0.275	0.01
	STEM	25.12	1	27.133	0	0.275	0.048
	Sex–STEM	1.76	1	1.901	0.168	0.275	0.003
	Residuals	503.646	544	NA	NA	0.275	NA
STEM-related gender stereotype	Int	0.012	1	0.013	0.909	0.323	NA
	Age	0.843	1	0.908	0.341	0.323	0.002
	Sex	39.575	1	42.626	0	0.323	0.073
	STEM	0.305	1	0.329	0.567	0.323	0.001
	Sex–STEM	0.001	1	0.001	0.973	0.323	0
	Residuals	505.063	544	NA	NA	0.323	NA
Educational aspirations in math	Int	0.408	1	0.423	0.516	0.287	NA
	Age	7.227	1	7.504	0.006	0.287	0.014
	Sex	5.467	1	5.677	0.018	0.287	0.01
	STEM	6.121	1	6.356	0.012	0.287	0.012
	Sex–STEM	0.526	1	0.546	0.46	0.287	0.001
	Residuals	523.914	544	NA	NA	0.287	NA
Friends’ attitudes towards math	Int	0.15	1	0.15	0.699	0.8	NA
	Age	0.005	1	0.005	0.944	0.8	0
	Sex	0.562	1	0.562	0.454	0.8	0.001
	STEM	3.216	1	3.218	0.073	0.8	0.006
	Sex–STEM	0.003	1	0.003	0.954	0.8	0
	Residuals	543.562	544	NA	NA	0.8	NA

**Table A4:** ANCOVA POST HOC results.

Variable	Group 1	Group 2	Diff	Adjusted *p*-Value
Ability self-perceptions in math	Female STEM	Female No-STEM	0	1
	Male No-STEM	Female No-STEM	0.01	1
	Male No-STEM	Female STEM	0.01	1
	Male STEM	Female No-STEM	0.24	>0.01 **
	Male STEM	Female STEM	0.24	>0.01 **
	Male STEM	Male No-STEM	0.23	0.03 *
Ability self-perceptions in science	Female STEM	Female No-STEM	−0.12	0.53
	Male No-STEM	Female No-STEM	−0.09	0.82
	Male No-STEM	Female STEM	0.03	0.99
	Male STEM	Female No-STEM	0.09	0.66
	Male STEM	Female STEM	0.21	0.04 *
	Male STEM	Male No-STEM	0.18	0.22
Self-perception of academic ability	Female STEM	Female No-STEM	−0.13	0.17
	Male No-STEM	Female No-STEM	−0.01	1
	Male No-STEM	Female STEM	0.12	0.32
	Male STEM	Female No-STEM	0.05	0.81
	Male STEM	Female STEM	0.18	>0.01 **
	Male STEM	Male No-STEM	0.06	0.81

Note: * *p* < 0.05. ** *p* < 0.01.

**Table A5:** Node statistics.

	Variable	Degree	Strength	Betweeness Centrality	Clustering Coefficient
STEM group	Spatial ability	4	0.15	0	0.33
	Self-perception of academic ability	5	1.17	0.35	0.3
	Intrinsic values (motivation)	6	1.2	0.27	0.33
	Intrinsic Motivation for math	6	0.88	0	0.4
	Ability self-perceptions in math	5	1.06	0.18	0.4
	Ability self-perceptions in science	3	0.93	0.29	0
	Intrinsic Motivation for science	4	1	0.35	0
	Non-verbal intelligence	4	0.24	0	0.17
	Perceived difficulty of math	4	0.68	0.13	0.5
	STEM-related gender stereotype	3	0.19	0	0.33
	Educational aspirations in math	6	0.36	0.02	0.53
	Friends’ attitudes towards math	4	0.21	0	0.5
No-STEM group	Spatial ability	4	0.33	0.04	0.67
	Self-perception of academic ability	3	1.16	0.2	0
	Intrinsic values (motivation)	5	1.32	0.15	0.3
	Intrinsic Motivation for math	7	1.11	0.13	0.38
	Ability self-perceptions in math	4	0.98	0.09	0.17
	Ability self-perceptions in science	5	0.96	0.18	0.3
	Intrinsic Motivation for science	4	0.87	0	0.17
	Non-verbal intelligence	3	0.21	0	1
	Perceived difficulty of math	6	0.62	0.07	0.4
	STEM-related gender stereotype	7	0.34	0.09	0.38
	Educational aspirations in math	4	0.46	0.02	0.67
	Friends’ attitudes towards math	4	0.27	0	0.67
Female group	Spatial ability	7	0.35	0.04	0.48
	Self-perception of academic ability	5	1.32	0.18	0.5
	Intrinsic values (motivation)	5	1.27	0.05	0.5
	Intrinsic Motivation for math	8	1.19	0.2	0.43
	Ability self-perceptions in math	6	1.07	0.22	0.53
	Ability self-perceptions in science	7	1.12	0.15	0.52
	Intrinsic Motivation for science	6	1.06	0.07	0.47
	Non-verbal intelligence	4	0.32	0	0.83
	Perceived difficulty of math	7	0.79	0.05	0.57
	STEM-related gender stereotype	7	0.33	0	0.62
	Educational aspirations in math	4	0.54	0.02	0.67
	Friends’ attitudes towards math	6	0.32	0	0.47
Male group	Spatial ability	7	0.39	0	0.29
	Self-perception of academic ability	4	1.2	0.25	0.5
	Intrinsic values (motivation)	6	1.25	0.2	0.2
	Intrinsic Motivation for math	6	1.04	0.13	0.2
	Ability self-perceptions in math	6	1.2	0.13	0.27
	Ability self-perceptions in science	4	0.99	0.29	0.17
	Intrinsic Motivation for science	5	1.06	0.33	0.1
	Non-verbal intelligence	6	0.31	0	0.33
	Perceived difficulty of math	5	0.65	0.02	0.6
	STEM-related gender stereotype	4	0.29	0.04	0.33
	Educational aspirations in math	5	0.35	0.02	0.3
	Friends’ attitudes towards math	4	0.26	0.02	0.33
